# The Origin of Cancer

**Published:** 1903-12-19

**Authors:** 


### The Origin of Cancer.

The delivery of Mr. Morris's Bradshaw Lecture before the Royal College of Surgeons of England, on the 9th instant, may be said to mark an epoch in the history of cancer investigation. His official positions as Treasurer of the Cancer Research Fund, and as Chairman of the Cancer Investigation Committee of the Middlesex Hospital, place him, as it were, upon a watch-tower, from which he is able at once to look back over the work which has been accomplished, and forward towards that which still remains to be done. It has now become necessary, he declares, to accept, at least provisionally and as a basis for further inquiry, the hypothesis of Cohnheim that tumours of all descriptions originate in the irregular germination and growth of scattered embryonic cells. The extent to which undeveloped embryonic cells must be regarded as forming part of the normal bodily structure, the localities in which they may be expected to occur, and the circumstances which tend to call them into activity, would appear to be the questions now calling for investigation; and these offer large opportunities alike to the embryologist and to the pathologist.

The conditions laid down by Mr. Morris, as probably or certainly tending to the development of tumours, are mainly a diminished vitality of the tissues surrounding the centre of morbid growth, and the presence of some influence by which that centre is subjected to irritation. In cancer of the lip, and in the cancer of chimney-sweeps, the local irritation is manifest, and it is perhaps hardly less so in cancers of the breast, of the uterus, or of the pylorus. There remain many cases in which no local cause of this kind is readily traceable, and in these it is reasonable to search for the consequences of chemical changes in the blood or tissues, such as may possibly be induced by excessive quantity or improper quality of food. The coincidence between a large consumption of food and an increase of cancer is too remarkable to be overlooked; and, although it may possibly be no more than a coincidence, it none the less calls for inquiry, and must be duly considered in any investigation which aims at being exhaustive. Another modern dietetic influence is that of the chemical adulteration of food by so-called preservatives, a development of "commerce" which may very likely be responsible for much unsuspected evil, and the effects of which should certainly be made the subjects of strict examination. Beyond these and other influences possibly operative upon the individual, we have the still larger possibilities of influences operative upon the race, and transmissible in their consequences to future generations. If morbid growths have their origins in scattered embryonic cells, are these cells to be found in all persons in approximately equal proportion, or are they more abundant in some than in others? Do they offer any indications of their presence, either normally or in excess, which can be regarded as danger signals, and can be utilised for the purpose of securing safety? Is their presence in excess, or in positions entailing an unusual amount of danger, a peculiarity likely to be transmitted to offspring, and thus to lay the foundation of an inherited diathesis? On all such points as these, and on many others which will from time to time suggest themselves, nothing can be ascertained by any process of guessing, however ingenious it may be. The case is one for the application of John Hunter's famous advice to Jenner, "Do not think, try." The means of trial, under competent guidance and on a scale of sufficient magnitude, are for the present at least being afforded by the Cancer Research Fund, and especially by the recent augmentation of its resources due to the enlightened liberality of Mr. Astor, but it must be remembered that these resources are not yet such as to place it in a position of permanent security against interruption of the good work in which it is engaged. The present main direction of the inquiry is into the effects of external conditions and of heredity upon many generations of cancer-prone animals; and its prosecution requires a combination of such patience and accuracy as only philosophers can exercise with such facilities in the way of funds and opportunities as only the public can supply. In the meanwhile, someone has been writing to the newspapers to protest against wholly imaginary "atrocities" supposed to be committed in what he calls "our" hospitals. The cackling of geese once afforded safety to Rome, and it is possible that the voice of this particular *anser* may usefully call attention to the merits and the claims of the Cancer Research Fund.

